# Feasibility of Atezolizumab in Combination With Chemotherapy for Children With Relapsed or Refractory Solid Tumors

**DOI:** 10.1002/pbc.32046

**Published:** 2025-09-12

**Authors:** Matthew E. Campbell, Sonja Stutzman, Sharon Primeaux, Deseray Sida, Minjae Lee, Avanthi T. Shah, Arhanti Sadanand, Elizabeth Sokol, Natalie B. Collins, Brian Turpin, Shoba Navai, Katie Albert, Theodore W. Laetsch, Dinesh Rakheja, Kenneth S. Chen, David E. Gerber, Andrew Y. Koh

**Affiliations:** 1 Division of Hematology/Oncology, Department of Pediatrics, University of Texas Southwestern Medical Center, Dallas, Texas, USA; 2 Department of Health Data Science & Biostatistics, Peter O’Donnell Jr. School of Public Health, University of Texas Southwestern Medical Center, Dallas, Texas, USA; 3 Division of Hematology, Oncology, and Stem Cell Transplantation, Department of Pediatrics, Ann and Robert H. Lurie Children’s Hospital of Chicago, Chicago, Illinois, USA; 4 Dana-Farber/Boston Children’s Cancer and Blood Disorders Center, Boston, Massachusetts, USA; 5 Pediatric Hematology/Oncology, Cincinnati Children’s Hospital Medical Center, Cincinnati, Ohio, USA; 6 Division of Hematology/Oncology, Department of Pediatrics, Baylor College of Medicine, Houston, Texas, USA; 7 Texas Children’s Hospital Cancer and Hematology Center, Houston, Texas, USA; 8 Division of Hematology/Oncology, Department of Pediatrics, University of Washington, Seattle, Washington, USA; 9 Division of Hematology/Oncology, Department of Pediatrics, University of Pennsylvania and Children’s Hospital of Philadelphia, Philadelphia, Pennsylvania, USA; 10 Department of Pathology, University of Texas Southwestern Medical Center, Dallas, Texas, USA; 11 Division of Hematology/Oncology, Department of Internal Medicine, University of Texas Southwestern Medical Center, Dallas, Texas, USA; 12 Harold C. Simmons Comprehensive Cancer Center, University of Texas Southwestern Medical Center, Dallas, Texas, USA; 13 Department of Microbiology, University of Texas Southwestern Medical Center, Dallas, Texas, USA

**Keywords:** combination therapy, immunotherapy, phase I trial, sarcoma, solid tumors

## Abstract

**Background:**

Combining immune checkpoint inhibitors (ICI) with chemotherapy may improve treatment response in children with solid tumors. We sought to determine the feasibility of combining vincristine, irinotecan, and temozolomide with the ICI atezolizumab in children with relapsed or refractory solid tumors (VITAS;).

**Methods:**

Patients ≥6 months and ≤18 years old with a relapsed or refractory solid tumor, no prior ICI, and evaluable disease per RECIST v1.1 were eligible for the Phase I cohort (NCT04796012). Patients received atezolizumab 15 mg/kg on Day 1, vincristine 1.5 mg/m^2^ on Day 1, irinotecan 50 mg/m^2^ on Days 1–5, and temozolomide 100 mg/m^2^ on Days 1–5 in 21-day cycles. The primary endpoint was the number of patients with dose-limiting toxicities (DLT) in the first two cycles of therapy.

**Results:**

Six patients (median age: 14 years) with rhabdomyosarcoma (*n* = 3), osteosarcoma (*n* = 2), and Ewing sarcoma (*n* = 1) received therapy and were evaluable for toxicity. Patients received a median of seven (range: 2–20) cycles of treatment. No patients experienced a DLT. One patient experienced Grade 2 immune-related colitis. Four patients experienced Grade ≥3 adverse events (decreased neutrophil count, febrile neutropenia, weight loss, anorexia). One patient with rhabdomyosarcoma had a sustained partial response through 16 cycles. One patient with relapsed pulmonary osteosarcoma has ongoing stable disease through 20 cycles.

**Conclusions:**

Atezolizumab combined with vincristine, irinotecan, and temozolomide was feasible and well tolerated in children with solid tumors. Efficacy of this regimen is now being assessed in relapsed and refractory rhabdomyosarcoma in an ongoing Phase II cohort.

## 1 | INTRODUCTION

Children with relapsed or refractory solid tumors have poor outcomes and limited treatment options. While immune checkpoint inhibitor (ICI) therapy has revolutionized the care of multiple adult cancers, it has shown limited monotherapy efficacy in pediatric solid tumors [1–3]. Potential reasons for this disparity include lower mutational burden in pediatric tumors, immature immune systems in pediatric populations, and distinct gut microbiomes [4–11].

Combining ICI with chemotherapy may improve these outcomes. Cytotoxic chemotherapy exposes tumor antigens and can create an immunogenic tumor microenvironment [12–17]. Commonly used chemotherapies also induce immune-mediated antitumor responses that, in turn, may be amplified by ICI [18]. Indeed, in multiple adult cancers, the combination of conventional chemotherapy with ICI has improved treatment response and has become standard of care [19–23]. It remains unknown whether that success can be extended to a pediatric population.

Atezolizumab is an ICI that blocks PD-L1 interaction with PD-1 and B7–1 on T cells to enhance tumor-specific responses and antitumor activity [24, 25]. Atezolizumab has demonstrated efficacy across multiple malignancies (both as monotherapy and in combination with chemotherapy), earning FDA approval for the treatment of non-small-cell lung cancer, small-cell lung cancer, hepatocellular carcinoma, and melanoma. Adverse reactions to atezolizumab include infusion-related reactions and various immune-related toxicities; these events have generally been manageable through treatment interruption or direct management of adverse reactions. When used in combination with chemotherapy in adults, toxicities have remained consistent with those of individual agents [21, 26, 27]. Although not approved for pediatric use, atezolizumab is well-tolerated in children at 15 mg/kg (maximum 1200 mg intravenously [IV] every 21 days), with a toxicity profile similar to that observed in adults [2].

The combination of vincristine, irinotecan, and temozolomide (VIT) is well-tolerated and has clinical activity in multiple tumor types, including relapsed or refractory rhabdomyosarcoma [28–30]. Further, VIT may promote antitumor immune responses through selectively depleting inhibitory regulatory T cells, increasing tumor mutational burden, and increasing tumor antigen presentation [12–17].

VITAS (NCT04796012) is a Phase I/II open-label, single-arm, multi-center study designed to assess the safety, feasibility, and efficacy of combining VIT with the ICI atezolizumab in children with relapsed or refractory solid tumors. Herein, we report the Phase I feasibility results and the recommended Phase II dosing of this novel combination.

## 2 | METHODS

### 2.1 | Objectives

The primary objective of the VITAS Phase I cohort was to determine the feasibility of administering vincristine, irinotecan, temozolomide, and atezolizumab simultaneously in children with relapsed or refractory solid tumors. The feasibility endpoint was defined as the number of patients with dose-limiting toxicities (DLT) in the first two cycles of therapy.

### 2.2 | Patient Eligibility

Patients aged ≥6 months and ≤18 years with a relapsed or refractory solid tumor (after at least one line of systemic therapy) were eligible for the Phase I feasibility cohort. Additional eligibility criteria included evaluable disease per RECIST v1.1; available tumor specimen (either from initial diagnosis or from a recurrence) to determine PD-L1 status; and performance status ≥50 per Lansky Performance Status (patients <16 years old) or Karnofsky Performance Status (patients ≥16 years old). Patients must have fully recovered from the acute toxic effects of all prior therapy and could not have received myelosuppressive therapy within 21 days of starting treatment, nonmyelosuppressive therapy within 7 days of start of protocol therapy, monoclonal antibodies within three half-lives of protocol therapy, cellular therapies within 28 days of protocol therapy, or major surgical procedure within 30 days of start of protocol therapy. Organ function requirements included absolute neutrophil count ≥1000/mm^3^, absolute lymphocyte count ≥500/mm^3^, hemoglobin ≥9 g/dL, platelets ≥75,000/mm^3^, total bilirubin ≤1.5× institutional upper limit of normal (ULN), ALT and ALT ≤2.5× ULN, serum albumin ≥25 g/L, creatinine ≤1.5× ULN for age or a creatinine clearance ≥70 mL/min/1.73 m^2^, INR or aPTT ≤1.5× ULN, and left ventricular ejection fraction ≥50% or shortening fraction ≥30%.

Exclusion criteria included pregnancy or breastfeeding, known allergy or hypersensitivity to any component of the study medications, history of severe allergic anaphylactic reaction to chimeric or humanized antibodies or fusion proteins, uncontrolled intercurrent illnesses including ongoing or untreated infections, prior ICI therapy, ongoing treatment with other investigational agents, other concomitant anticancer agents, systemic immunostimulatory agents, and diagnosis of an autoimmune disorder.

### 2.3 | Study Design and Treatment Schema

This study (NCT04796012) was opened at five pediatric cancer centers. UT Southwestern was the coordinating center. The study was approved by the UT Southwestern Single Institutional Review Board (IRB; STU-2021–0606) for all sites. Written informed consent and assent were obtained according to institutional guidelines.

**FIGURE 1 |:**
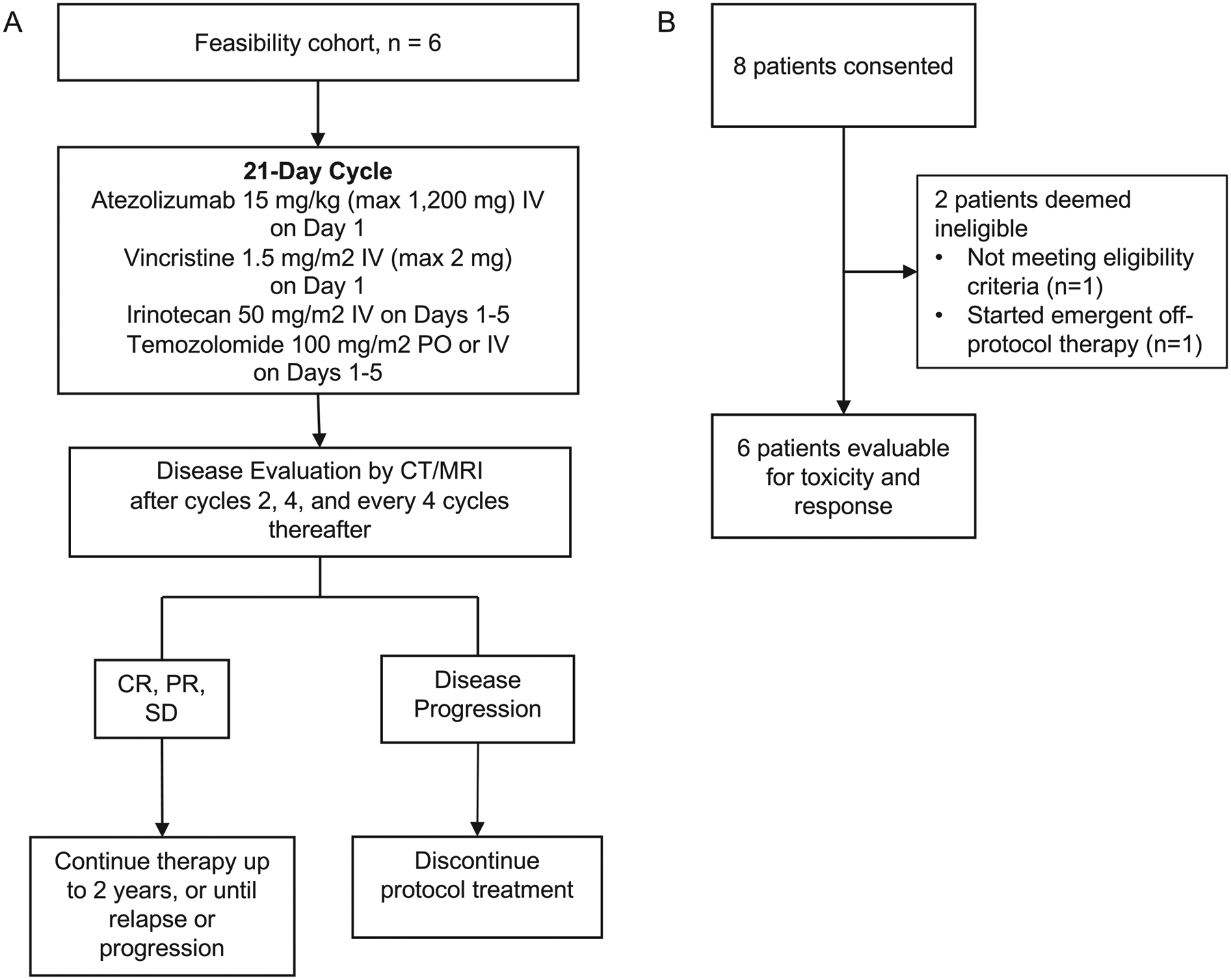
Trial design. (A) Phase I/II design with cohort recruitment, treatment plan, and disease evaluation plan. (B) Phase I trial profile accounting for all patients who consented to the study. CR, complete response; PR, partial response; SD, stable disease.

Treatment was administered in 21-day cycles (Figure 1). Patients received atezolizumab 15 mg/kg (maximum 1200 mg) on Day 1 of each 21-day cycle, vincristine 1.5 mg/m^2^ (maximum 2 mg) on Day 1, irinotecan 50 mg/m^2^ IV on Days 1–5, and temozolomide 100 mg/m^2^ on Days 1–5 (orally [PO] or IV). Subsequent cycles were administered if the patient had no clinical or radiographic evidence of progressive disease, adequate laboratory parameters (including unsupported platelet count ≥75,000/mm^3^ and absolute neutrophil count ≥750/mm^3^), and recovery from treatment-related toxicities to an acceptable grade per protocol. If patients experienced an atezolizumab-related adverse event requiring active treatment with steroids at a dosing equivalent of prednisone ≥10 mg/day, atezolizumab was withheld, and the other study drugs could be administered. Patients were removed from the study if atezolizumab needed to be held for two consecutive cycles.

Patients could receive local therapy (radiation or surgery) to non-target lesions after at least four cycles of atezolizumab, provided it did not interfere with the assessment of tumor target lesions. Patients did not receive antibiotic prophylaxis for irinotecan-associated diarrhea, given that antibiotics are known to impact the efficacy of immune checkpoint inhibitor therapy [31]. The protocol guided the management of irinotecan-associated diarrhea with the specific recommendation to use atropine daily while administering irinotecan, regardless of patient symptoms. All patients received oral trimethoprim-sulfamethoxazole for *Pneumocystis jirovecii* prophylaxis while on protocol therapy.

Patients could receive treatment in the study for up to 2 years. Subjects were removed from protocol therapy for disease progression, intercurrent illness that prevented further administration of treatment, unacceptable adverse event(s), patient decision to withdraw from the study, or changes in the patient’s condition that rendered the patient ineligible for further treatment in the judgment of the investigator.

### 2.4 | Assessments

Common Terminology Criteria for Adverse Events (CTCAE) version 5.0 was used for toxicity grading. DLT evaluation occurred from Day 1 of Cycle 1 until Day 1 of Cycle 3 or 30 days from Day 1 of Cycle 2, whichever was earlier. Non-hematological DLTs were defined as any toxicity that resulted in a delay of a subsequent cycle by more than 14 days or withholding atezolizumab for ≥28 days, except allergic reactions, and Grade ≥3 toxicity possibly, probably, or definitely attributable to the investigational regimen. Specific exceptions included Grade 3 nausea and vomiting ≤3 days duration, Grade 3 mucositis or stomatitis ≤3 days duration, Grade 3 fever, Grade 3 infection, or Grade 3 hypophosphatemia, hypokalemia, hypocalcemia, or hypomagnesemia responsive to supplementation within 7 days. Hematological DLTs were defined as Grade 4 neutropenia for more than 7 days, Grade 4 anemia (unexplained by underlying malignancy), platelet count less than 20,000/mm^3^ on two separate days or requiring a platelet transfusion on 2 separate days within a 7-day period, or myelosuppression that caused a delay of more than 14 days between treatment cycles. Myeloid growth factors could not be used in Cycle 1, except in cases of documented infection. Guidance on the identification and treatment of immune-related adverse events (irAEs) was outlined in the study protocol.

Six patients were identified for accrual at the initial dose level. The treatment regimen was to be deemed feasible, and the recommended Phase 2 dose (RP2D) would be established if no more than two patients developed DLTs. If more than two patients developed a DLT, then doses would be de-escalated, depending on the types and attributions of toxicities exhibited. The feasibility cohort would then be extended until six patients were accrued at the same dose level.

Treatment response was determined per RECIST v1.1. Tumor assessments were performed within the last week of Cycles 2, 4, 8, and every four cycles thereafter. PD-L1 immunohistochemistry was performed in a central laboratory using the 22c3 antibody and interpreted by a board-certified pathologist (D.R.). Partial or complete membranous staining of any intensity was considered positive, and PD-L1 positivity was calculated as a combined positive score (CPS), defined previously as the sum of positive viable tumor cells and positive mononuclear inflammatory cells divided by the total number of viable tumor cells and the fraction then multiplied by 100 [32]. A CPS score ≥1 was considered positive, but was not required for eligibility, and was performed retrospectively after enrollment.

### 2.5 | Statistical Considerations

Demographic and clinical characteristics were summarized using descriptive statistics, including median and range for continuous variables and proportions and frequencies for categorical variables.

## 3 | RESULTS

Eight patients consented to participate and were screened (Figure 1). Two patients were excluded: one patient started emergent off-protocol chemotherapy, and one patient did not meet eligibility criteria. The remaining six patients received therapy per protocol and were evaluable for toxicity. The median age was 14 years (range: 8–16 years), and three patients (50%) were female (Table 1). Three patients had rhabdomyosarcoma, two patients had osteosarcoma, and one patient had Ewing sarcoma. Patients had received a median of three (range: 1–3) prior lines of systemic therapy.

Patients received a median of seven cycles (range: 2–20). One patient stopped therapy after Day 1 of Cycle 8 due to intolerable toxicity (Grade 2 nausea, Grade 3 anorexia). One patient received myeloid growth factor after Cycle 1, at least once during protocol therapy. Four patients received systemic antibiotics for fever at least once during protocol therapy. One patient received oral vancomycin for *Clostridium difficile* infection.

### 3.1 | Safety

All six treated patients received at least two cycles of protocol therapy and were included in toxicity analysis. No patients experienced a DLT. Overall, five patients (83%) experienced Grade 3 or 4 adverse events (AEs, Table 2), including decreased neutrophil count (*n* = 4, 67%), decreased white blood cell count (*n* = 1, 17%), febrile neutropenia (*n* = 1, 17%), anorexia (*n* = 1, 17%), and weight loss (*n* = 1, 17%). No patient had a treatment delay of more than 7 days due to Grade 3 or 4 toxicities.

The most common toxicities of all grades were nausea (*n* = 6, 100%), vomiting (*n* = 5, 83%), diarrhea (*n* = 5, 83%), anorexia (*n* = 5, 83%), and decreased neutrophil count (*n* = 4, 67%). All toxicities of all grades are shown in Table S1. One patient developed Grade 2 immune-related colitis in Cycle 4 that was confirmed on biopsy and pathologic review. This patient had resolution of diarrhea within 14 days, did not require treatment with steroids, and did not have a treatment delay due to colitis. No patients died in the study from treatment-related toxicity.

### 3.2 | Efficacy

**FIGURE 2 |:**
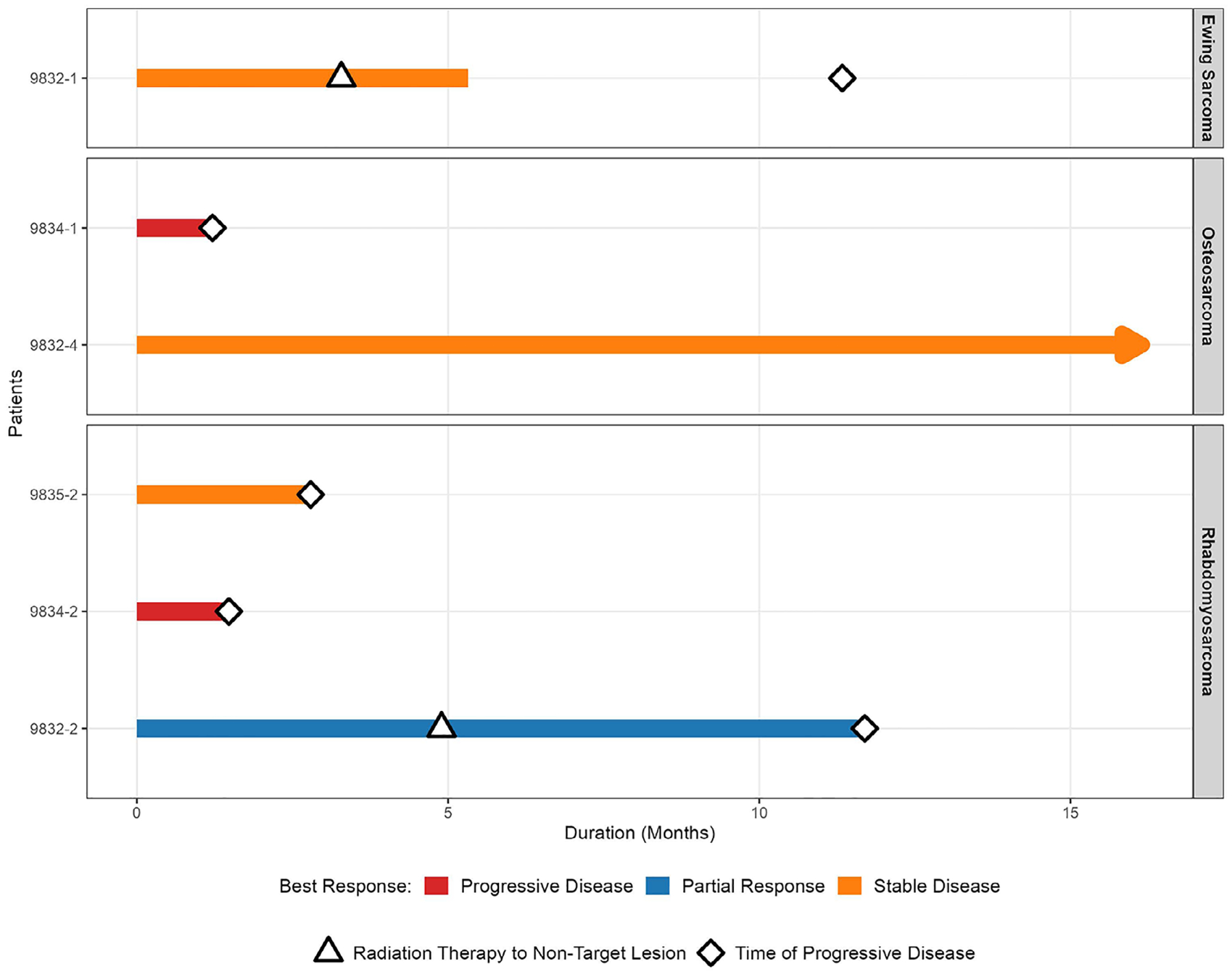
Swimmer’s plot of six patients treated in the study, with timing of events from Day 1 of treatment. Patient 9832–1 is represented as Stable Disease but had Evaluable Disease that was Non-Complete Response/Non-Progressive Disease.

All six enrolled patients received at least two cycles of protocol therapy and were eligible for assessment of disease response per RECIST v1.1 (Figure 2).

One patient with recurrent Ewing sarcoma of the bony pelvis had an incomplete response of evaluable disease with decreased PET avidity after four cycles of VITAS therapy. He subsequently received radiation to the site of disease while continuing protocol therapy and had complete resolution of PET avidity. The patient elected to come off protocol therapy after Day 1 of Cycle 8 due to intolerable side effects (nausea, anorexia). He experienced disease recurrence 6 months after stopping therapy.

Two patients had osteosarcoma with measurable disease. One patient with paraspinal disease had progressive disease after two cycles of therapy. The other patient had unresectable pulmonary disease and had stable disease through 20 cycles, and remains on therapy at the time of writing this report.

**FIGURE 3 |:**
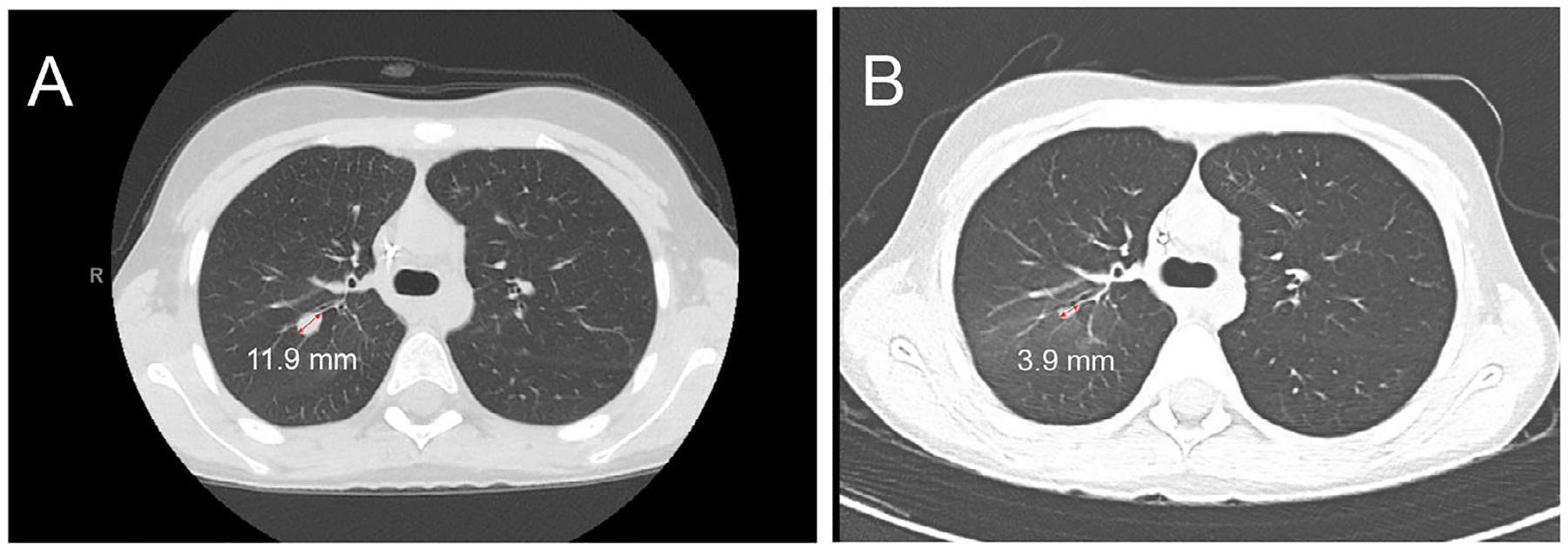
Serial CT chest images of patient 9832–2 with MYOD1-mutant spindle cell/sclerosing RMS who had a sustained PR to VITAS therapy through 16 cycles. Imaging at baseline (A) and after two cycles of protocol therapy (B). CT, computed tomography; RMS, rhabdomyosarcoma; PR, partial response.

Three patients had rhabdomyosarcoma with measurable disease. One patient with *FOXO1*-fusion-positive rhabdomyosarcoma with multiple sites of disease had stable disease after two cycles of therapy, but developed progressive disease after four cycles of therapy. One patient with FOXO1-fusion-positive rhabdomyosarcoma with paranasal and orbital disease had progressive disease after two cycles of therapy. One patient with *MYOD1*-mutant spindle cell/sclerosing rhabdomyosarcoma had a sustained partial response through 16 cycles of therapy, but had disease progression after 20 cycles of therapy (Figure 3). She received radiation to non-target lesions after four cycles of chemotherapy.

The PD-L1 CPS scores for all patients are listed in Table 3. Two patient tumors were PD-L1-positive: patient 9832–4 with pulmonary metastatic osteosarcoma with stable disease through 20 cycles had a CPS score of 5, and patient 9832–2 with *MYOD1*-mutant spindle cell/sclerosing rhabdomyosarcoma with a sustained partial response through 16 cycles had a CPS score of 9. All other patients had a CPS score less than 1.

## 4 | DISCUSSION

ICI monotherapy has limited efficacy in pediatric cancers. Combining ICI with chemotherapy is supported by strong biologic rationale and efficacy in numerous adult cancers. This study demonstrated the safety and feasibility of combining VIT with atezolizumab for children and adolescents with relapsed or refractory solid tumors. No patients experienced DLTs. While most patients experienced Grade ≥3 toxicity, all adverse events were anticipated, and importantly, none resulted in a treatment delay of more than 7 days.

All patients experienced nausea, and most experienced diarrhea. This was anticipated and monitored closely. Patients did not receive antibiotic prophylaxis for irinotecan-associated diarrhea, given that antibiotics are known to impact the efficacy of ICI therapy. Antibiotic prophylaxis is standard of care when administering irinotecan, but the safety of its use without prophylaxis has previously been demonstrated [33]. No patients experienced Grade 3 or 4 diarrhea, and no patients had treatment delays due to diarrhea. One patient experienced autoimmune toxicity (colitis) that resolved without steroids. As such, patients receiving this combination in Phase II will not receive antibiotic prophylaxis for irinotecan-associated diarrhea.

One patient with rhabdomyosarcoma had a sustained partial response through 16 cycles. One patient with osteosarcoma has had stable disease through 20 cycles of therapy. One patient with evaluable Ewing sarcoma did not experience disease progression while on protocol therapy. Interestingly, the patients with rhabdomyosarcoma and osteosarcoma who experienced apparent clinical benefit, both had a PD-L1 CPS score greater than 1. While anecdotal, these clinical data suggest potential value in further exploring the efficacy of this treatment combination. A Phase II rhabdomyosarcoma efficacy cohort was planned a priori, as the effectiveness of VIT in treating relapsed rhabdomyosarcoma in children has been well assessed, allowing for estimation of a baseline response rate [28–30]. Further, there is evidence that the PD-1/PD-L1 signaling axis is biologically relevant in rhabdomyosarcoma [34, 35]. Hence, our Phase II rhabdomyosarcoma efficacy cohort is currently enrolling patients. Osteosarcoma and Ewing sarcoma Phase II cohorts also merit consideration for future trials.

These results also highlight the importance of ensuring an adequate number of study patients have PD-L1(+) tumors. Indeed, our study requires at least eight of 17 patients in the rhabdomyosarcoma Phase II cohort to have PD-L1(+) tumors for adequate subset analysis. Future correlative studies of patient tumor, blood, and stool may also lend insights into characteristics associated with response to therapy.

Our study has several limitations. VITAS was a histology-agnostic Phase I study with a limited sample size. While the partial response observed in a patient with rhabdomyosarcoma and the durable stable disease observed in patients with Ewing sarcoma and osteosarcoma were encouraging, we cannot draw any definitive conclusions regarding treatment efficacy overall or in disease-specific cohorts. Future Ewing sarcoma or osteosarcoma Phase II expansion cohorts could provide valuable insight. Finally, the youngest patient was 8 years old, and most patients were adolescents, which, to some extent, limits the assessment of toxicities that may be observed in younger children (as our study was open to children as young as 6 months of age).

ICIs and their combination with chemotherapy have so far had a limited role in the treatment of pediatric cancers, primarily restricted to several lymphoma subtypes and solid tumors with high mutation burden or mismatch-repair deficiency [36]. To our knowledge, this is the first trial testing the specific combination of VIT, a safe and widely used chemotherapy backbone for relapsed solid tumors, with ICI therapy. This is also the first such trial with a specific focus on treatment effect in rhabdomyosarcoma (RMS).

In conclusion, combining atezolizumab with VIT to treat children with relapsed solid tumors was feasible at previously established doses with an expected and manageable toxicity profile. The combination showed promising disease control in one patient with rhabdomyosarcoma, one patient with Ewing sarcoma, and one with osteosarcoma. A more thorough assessment of antitumor activity is ongoing in a Phase II rhabdomyosarcoma cohort.

## Supplementary Material

Table S1 TABLE S1 All toxicities that were possibly, probably, or definitely attributed to therapy (n=6).

Additional supporting information can be found online in the Supporting Information section.

## Figures and Tables

**TABLE 1 |:** Demographics and patient characteristics.

Characteristics	Number (%)
Total number of evaluable patients	6
Age	
Median	14
Range	8–16
Sex	
Male	3 (50%)
Female	3 (50%)
Race	
Asian	1 (17%)
Black	1 (17%)
White	4 (67%)
Ethnicity	
Hispanic	1 (17%)
Non-Hispanic	5 (86%)
Diagnosis	
Rhabdomyosarcoma	3 (50%)
Osteosarcoma	2 (33%)
Ewing sarcoma	1 (17%)
Prior lines of therapy	
Median	3
Range	1–3
Cycles of VITAS therapy	
Median	4
Range	2–20

**TABLE 2 |:** Treated patient summary.

Patient #	Age (years)	Diagnosis	Prior lines of therapy	Disease status	PD-L1 CPS score	Best OR	DLT	Treatment course
9832–1	14	ES	1	Evaluable	<1	Non-CR/non-PD	No	7 complete cycles, off therapy Cycle 8, Day 2 due to patient preference
9832–2	8	RMS	3	Measurable	9	PR	No	20 cycles, off therapy due to PD
9832–4	18	OS	1	Measurable	5.2	SD	No	Continues therapy through Cycle 20 with SD
9834–1	14	OS	3	Measurable	<1	PD	No	2 cycles, off therapy due to PD
9834–2	15	RMS	1	Measurable	<1	PD	No	2 cycles, off therapy due to PD
9835–2	16	RMS	3	Measurable	<1	SD	No	4 cycles, off therapy due to PD

Abbreviations: CPS, combined positive score; CR, complete response; DLT, dose-limiting toxicity; ES, Ewing sarcoma; OS, osteosarcoma; PD, progressive disease; PR, partial response; RMS, rhabdomyosarcoma; SD, stable disease.

**TABLE 3 |:** Grade 3 and 4 toxicities possibly, probably, or definitely attributed to therapy (*n* = 6).

Type of toxicity	Common Terminology Criteria for Adverse Events grade number (%)	
	Grade 3	Grade 4
Blood and lymphatic system disorders		
Febrile neutropenia	1 (17%)	0 (0%)
Investigations		
Neutrophil count decreased	2 (33%)	2 (33%)
Weight loss	1 (17%)	0 (0%)
White blood cell count decreased	0 (0%)	1 (17%)
Metabolism and nutrition disorders		
Anorexia	1 (17%)	0 (0%)

## Data Availability

Research data are not shared.

## Abbreviations:

AE: adverse event
CPS: combined positive score
CTCAE: Common Terminology Criteria for Adverse Events
DLT: dose-limiting toxicity
ICI: immune checkpoint inhibitor
irAE: immune-related adverse events
RP2D: recommended Phase 2 dose
ULN: upper limit of normal
VIT: vincristine, irinotecan, temozolomide
