# Supplementary material for: Feasibility of Atezolizumab in Combination With Chemotherapy for Children With Relapsed or Refractory Solid Tumors
Source: Pediatr Blood Cancer. Author manuscript; Available in PMC 2026 Aug 6. (PMC13445394; doi:10.1002/pbc.32046)
Supplement: Supplementary file 1 [file NIHMS2194380-supplement-Table_S1.docx]

| **TABLE S1 All toxicities that were possibly, probably, or definitely attributed to therapy (n=6)** | | | | |
| --- | --- | --- | --- | --- |
| Type of Toxicity | Common Terminology Criteria for Adverse Events Grade  Number (%) | | | |
|  | Grade 1 | Grade 2 | Grade 3 | Grade 4 |
| Blood and lymphatic system disorders | | | | |
| Anemia | 0 (0%) | 2 (33%) | 0 (0%) | 0 (0%) |
| Fatigue | 0 (0%) | 1 (17%) | 0 (0%) | 0 (0%) |
| Febrile Neutropenia | 0 (0%) | 0 (0%) | 1 (17%) | 0 (0%) |
| Cardiac disorders | | | | |
| Sinus tachycardia | 0 (0%) | 1 (17%) | 0 (0%) | 0 (0%) |
| Gastrointestinal Disorders | | | | |
| Abdominal pain | 1 (17%) | 1 (17%) | 0 (0%) | 0 (0%) |
| Colitis | 0 (0%) | 1 (17%) | 0 (0%) | 0 (0%) |
| Constipation | 2 (33%) | 1 (17%) | 0 (0%) | 0 (0%) |
| Diarrhea | 3 (50%) | 2 (33%) | 0 (0%) | 0 (0%) |
| Dry Mouth | 1 (17%) | 0 (0%) | 0 (0%) | 0 (0%) |
| Gastritis | 0 (0%) | 1 (17%) | 0 (0%) | 0 (0%) |
| Nausea | 2 (33%) | 4 (67%) | 0 (0%) | 0 (0%) |
| Vomiting | 1 (17%) | 4 (67%) | 0 (0%) | 0 (0%) |
| General disorders and administration site conditions | | | | |
| Fatigue | 3 (50%) | 0 (0%) | 0 (0%) | 0 (0%) |
| Fever | 1 (17%) | 1 (17%) | 0 (0%) | 0 (0%) |
| Infections and infestations | | | | |
| Urinary Tract Infection | 0 (0%) | 1 (17%) | 0 (0%) | 0 (0%) |
| Injury, poisoning and procedural complications | | | | |
| Bruising | 1 (17%) | 0 (0%) | 0 (0%) | 0 (0%) |
| Infusion Related Reaction | 0 (0%) | 1 (17%) | 0 (0%) | 0 (0%) |
| Investigations | | | | |
| ALT Increased | 1 (17%) | 1 (17%) | 0 (0%) | 0 (0%) |
| AST increased | 1 (17%) | 0 (0%) | 0 (0%) | 0 (0%) |
| Lipase Increased | 1 (17%) | 0 (0%) | 0 (0%) | 0 (0%) |
| Lymphocyte count decreased | 0 (0%) | 1 (17%) | 0 (0%) | 0 (0%) |
| Neutrophil count decreased | 0 (0%) | 0 (0%) | 2 (33%) | 2 (33%) |
| Platelet Count Decreased | 1 (17%) | 1 (17%) | 0 (0%) | 0 (0%) |
| Serum amylase increased | 1 (17%) | 1 (17%) | 0 (0%) | 0 (0%) |
| Weight loss | 1 (17%) | 1 (17%) | 1 (17%) | 0 (0%) |
| White blood cell count decreased | 0 (0%) | 1 (17%) | 0 (0%) | 1 (17%) |
| CRP elevated | 1 (17%) | 0 (0%) | 0 (0%) | 0 (0%) |
| Metabolism and nutrition disorders | | | | |
| Anorexia | 2 (33%) | 2 (33%) | 1 (17%) | 0 (0%) |
| Constipation | 1 (17%) | 0 (0%) | 0 (0%) | 0 (0%) |
| Dehydration | 0 (0%) | 1 (17%) | 0 (0%) | 0 (0%) |
| Hyperglycemia | 1 (17%) | 0 (0%) | 0 (0%) | 0 (0%) |
| Hypoalbuminemia | 1 (17%) | 0 (0%) | 0 (0%) | 0 (0%) |
| Hypokalemia | 2 (33%) | 0 (0%) | 0 (0%) | 0 (0%) |
| Hyponatremia | 1 (17%) | 0 (0%) | 0 (0%) | 0 (0%) |
| Nervous system disorders | | | | |
| Headache | 1 (17%) | 0 (0%) | 0 (0%) | 0 (0%) |
| Skin and subcutaneous disorders | | | | |
| Alopecia | 1 (17%) | 3 (50%) | 0 (0%) | 0 (0%) |
| Dry skin | 1 (17%) | 0 (0%) | 0 (0%) | 0 (0%) |
